# Mouse models of gastric preneoplasia and cancer, with a focus on pathology

**DOI:** 10.1242/dmm.052902

**Published:** 2026-10-28

**Authors:** Raymond Ho, Yongji Zeng, Jason C. Mills

**Affiliations:** ^1^ Department of Molecular and Precision Medicine, Penn State College of Medicine, Hershey, PA 17033, USA; ^2^ Medical Scientist Training Program, Baylor College of Medicine, Houston, TX 77030, USA

**Keywords:** Gastric cancer, Mouse models, SPEM, Intestinal metaplasia, Pseudoinvasion, Paligenosis

## Abstract

Gastric cancer is the fifth most diagnosed cancer and the fifth leading cause of cancer deaths worldwide, with limited options of targeted therapy available. Thus, a better understanding of key molecular events that contribute to gastric tumorigenesis would facilitate the development of novel therapies to prevent or treat gastric cancer. Extensive efforts have been made towards generating murine models that recapitulate the complex process of gastric cancer pathogenesis. There have been some challenges, however, in identifying and interpreting the results obtained by using these models. Namely, hyperplasias have often been misinterpreted as invasive cancer, and identifying cancer cells of origin has been complicated by nonspecific lineage-tracing studies. In this Review, we summarize the current models of gastric preneoplasia and cancer and discuss their limitations, especially with regard to correlating mouse and human pathology.

## Teaser

**Summary:** This Review summarizes mouse models of preneoplasia and cancer of the stomach, with a particular focus on the interpretation of the histopathology observed in these models.

## INTRODUCTION

Gastric cancer is the fifth most diagnosed cancer and the fifth leading cause of cancer deaths worldwide, with nearly 1 million new cases documented in 2022 (Bray et al., 2024). The high mortality is believed to stem from late-stage diagnosis, because risk of death by gastric cancer is reduced by nearly half in countries in which screening to detect early lesions is common (Kim et al., 2018). Although effective interventions such as surgery and chemoradiotherapy are available, they are associated with significant complications. For example, surgical resection causes serious morbidity in ∼25% of patients with advanced disease (Papenfuss et al., 2014). In addition, it is associated with reduced quality of life, even for patients with early-stage disease at the time of intervention (Eom et al., 2024; Gallanis et al., 2024). Thus, there remains a critical need for novel targeted therapy options that provide improved patient outcomes (Joshi and Badgwell, 2021). Targeted therapies for gastric cancer, although so far scarce and showing modest efficacy in clinical trials (Patel and Cecchini, 2020; Nakamura et al., 2021; Zeng and Jin, 2022), have the potential to change the treatment paradigm by eliminating the need for surgery or by preventing progression of precancerous lesions (Goldenring et al., 2025). The development of such therapies requires a thorough understanding of the molecular pathogenesis of gastric cancer, which is complicated by its genetically heterogeneous nature (Wong et al., 2014; Ho and Tan, 2019; Hess et al., 2023). Murine models have helped uncover the molecular mechanisms underlying numerous cancers, with such models being ever more rapidly developed since the advent of CRISPR/Cas9 technology (Walrath et al., 2010; Heyer et al., 2010; Kersten et al., 2017; Lampreht Tratar et al., 2018). For example, widely adopted murine models of pancreatic adenocarcinoma involving mutations in the most common cancer driver, *Kras*, are rapidly advancing understanding of pancreatic tumorigenesis (Guerra and Barbacid, 2013). However, the field of mouse models of gastric cancer has been hindered by a lack of consensus on pathways and mutant genes involved, which may reflect the heterogeneity of gastric cancer in humans (Hess et al., 2023). Moreover, technical issues with some of these models have resulted in instances of misinterpretation of the cell of origin for gastric cancer. In addition, there has been confusion in defining what constitutes invasive cancer in these models (see Box 1). Here, we seek to review and provide perspective on the current state of mouse models for gastric preneoplasia and gastric cancer, and address some of these challenges.

Box 1. Caveat in the pathological interpretation of many published mouse models of gastric cancer Mice are known to develop gastric cancer of both intestinal and diffuse/signet-ring types, and both types can be locally invasive and metastasize to the liver. In addition, the mouse stomach, with its relatively thin submucosa and muscularis mucosa, can also exhibit submucosal infoldings and invaginations, especially in the presence of exuberant epithelial proliferation (Fig. 4). However, these pseudo-invasive foci are not truly invasive cancer. They likely arise because of buckling of the tissue section, such that the bases of the tortuous, hyperplastic glands cut in cross-section and muscularis mucosa appears to surround the focus (Fig. 4). Sometimes, the epithelium does seem to invaginate into the submucosa, but the invaginating glands lack the directionality of invading cancer cells. The glands in these cases are not irregular or asymmetrical, and the glandular cells do not exhibit polarity defects or changes in nuclear conformation or other signs of cellular atypia characteristic of cancer. Thus, in our opinion, there is considerable overcalling of invasive cancer in publications on mouse cancer models.

Similarly, there is considerable overcalling of ‘intestinal metaplasia’ in mouse models. Many publications have interpreted Alcian Blue staining of gastric epithelial cells as intestinal metaplasia, even though it also stains normal mucous neck and deep antral mucous cells in the stomach. The Alcian Blue staining is light in uninjured tissue, and its intensity increases in gastric epithelia cells during inflammation, particularly in spasmolytic-polypeptide-expressing metaplasia cells and in mucous neck cell hyperplasia. If intestinal metaplasia in mouse stomach is used to model human intestinal metaplasia, it must also exhibit characteristic morphology, including goblet and/or Paneth cells and/or enterocytes with characteristic terminal bars due to apical microvilli and their actomyosin cytoskeletal anchors. This can be confirmed by CDX2 nuclear staining (CDX2 does not label gastric-type epithelial cells) or MUC2 in cells with goblet morphology. Such human-like intestinal metaplasia is exceedingly rare in mouse models analyzed to date.

## Classification and progression of gastric cancer

According to Lauren classification (Lauren, 1965), gastric cancer has been classically divided into two broad histological subtypes: intestinal and diffuse, with the intestinal subtype being more common worldwide (Sitarz et al., 2018). However, genetic subtyping of gastric cancer, facilitated by the development of modern sequencing and omics technologies, has enabled more granular classification (Wang et al., 2025), with substantial input from such groups as The Cancer Genome Atlas (TCGA) Research Network (The Cancer Genome Atlas Research Network, 2014) and the Asian Cancer Research Group (ACRG) (Cristescu et al., 2015). TCGA identified four subtypes of gastric cancer: Epstein–Barr virus (EBV)-positive, microsatellite unstable, genomically stable and chromosomally unstable (The Cancer Genome Atlas Research Network, 2014). The ACRG identified four other subtypes: mesenchymal-like, microsatellite unstable, TP53 active and TP53 inactive (Cristescu et al., 2015). Molecular characterization has identified genes that are frequently mutated in these subtypes, including *CDH1*, *KRAS*, *TP53*, *APC* and *ARID1A* (The Cancer Genome Atlas Research Network, 2014; Cristescu et al., 2015). Although some subtypes are relatively similar, others are quite different, indicating that the clinical application of molecular subtyping for prognosis and therapy is still evolving. Nevertheless, identification of these molecular subtypes and the specific genes altered has helped guide the development of corresponding murine models to recapitulate human disease.

Correa famously posited that intestinal-type gastric cancer develops through a stereotypical sequence: chronic gastritis, oxyntic atrophy, intestinal metaplasia and dysplasia (Correa, 1988, 1992) (Fig. 1). In most cancers originating via this sequence, the initial chronic gastritis and consequent oxyntic atrophy are caused by infection with the bacterium *Helicobacter pylori* (Fox and Wang, 2007; Liabeuf et al., 2022). Oxyntic atrophy is defined by loss of the acid-secreting parietal cells of the stomach and correlates with emergence of altered organization of the whole gastric unit in a pattern known as pyloric or pseudopyloric metaplasia (Nozaki et al., 2008; Petersen et al., 2017). In pyloric metaplasia, basal, zymogen-secreting chief cells are replaced by mucinous cells that express markers such as TFF2 (formally known as spasmolytic polypeptide), MUC6, AQP5 and CD44v9 (Cornish et al., 2026). Numerous lines of evidence in non-human animals have shown that such spasmolytic-polypeptide-expressing metaplasia (SPEM) cells – which molecularly and morphologically phenocopy deep glandular mucous cells of the stomach antrum – largely arise via reprogramming of the chief cells (Goldenring et al., 2010). Human tissue shows identical stages of conversion, indicating that chief cells are also the main source of SPEM cells in humans (Lennerz et al., 2010; Mills and Sansom, 2015; Mills and Goldenring, 2017; Goldenring and Mills, 2022). The chief cell-to-SPEM cell conversion sequence (as well as a return of SPEM to chief cells if inflammation subsides) has been studied in detail for well over a century (Harvey, 1906; Smith, 1916; Weis and Goldenring, 2009; Meyer and Goldenring, 2018). Evidence suggests that this process represents a normal developmental pattern, as the cardiac mucosa may arise embryonically through oxyntic glands in the fundus, losing parietal cells and having chief cells switch to a mucinous, deep pyloric gland phenotype (i.e. a SPEM-like pattern) (Kirk, 1907; Karam and Leblond, 1995; Goldenring et al., 2011; Kim and Shivdasani, 2016).

**Fig. 1.**
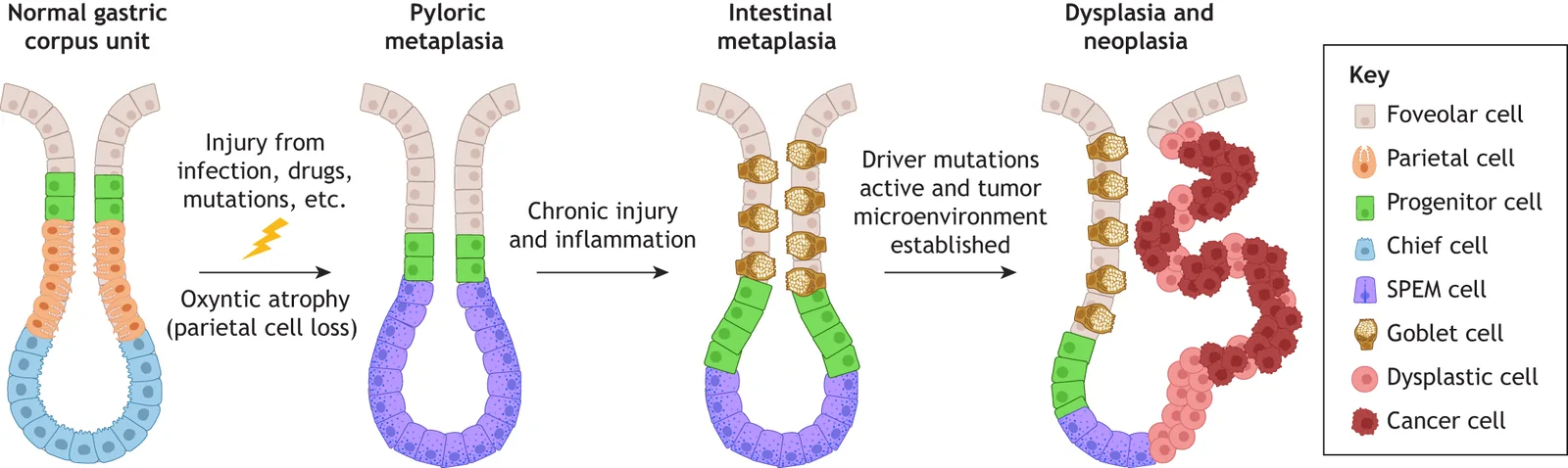
**Stages of gastric cancer progression.** Gastric tumorigenesis is thought to proceed in a stepwise process. First, injury to the stomach causes gastritis and oxyntic atrophy, resulting in the development of pyloric metaplasia, characterized by foveolar cell expansion and paligenosis of chief cells into proliferating spasmolytic-polypeptide-expressing metaplasia (SPEM) cells. Pyloric metaplasia is normally reversible; however, when the injury and/or inflammation becomes chronic, it can then progress to intestinal metaplasia, which features the abnormal presence of goblet cells in the stomach corpus. The activation of multiple driver mutations and influence from the microenvironment in intestinal metaplasia, particularly in its incomplete form defined by mixed gastric and intestinal phenotypes, results in further progression to dysplasia and, finally, neoplasia. Created in BioRender by Zeng, Y. (2026). https://BioRender.com/zhgecln. This figure was sublicensed under CC-BY 4.0 terms.

In humans, pyloric metaplasia is thought to be a temporary, regenerative state (Goldenring and Mills, 2022). However, as chronic gastritis continues and this state does not resolve, cells with intestinal differentiation can emerge. Such intestinal metaplasia occurs in so-called complete and incomplete forms (Goldenring and Mills, 2022; Kim et al., 2025). Considerable epidemiological and molecular–cellular evidence has implicated incomplete intestinal metaplasia as carrying higher risk for progression to neoplasia versus complete intestinal metaplasia (Goldenring and Mills, 2022; Tong et al., 2024). Complete intestinal metaplasia is defined by the full transformation of gastric epithelial units, from the lumen to the base, into a small-intestine-like organization with multiple mature intestinal cells present, including mature goblet and Paneth cells (Wong et al., 2000; Huang et al., 2019), and features markers including CDX2 and MUC2 (Liu et al., 2007). Incomplete intestinal metaplasia is defined by mixed gastric and intestinal phenotype with transitional/indeterminate cells that show, for example, only partial goblet cell differentiation (Correa and Piazuelo, 2012), and features markers including MUC2, MUC5AC and MUC6 (Babu et al., 2006). The deepest (basal) cells in incomplete intestinal metaplasia are typically, uniformly SPEM-like, similar to pyloric metaplasia [see fig. 1 of Cornish et al. (2026) and Kim et al. (2025)].

## Preneoplasia models

Although there has been great progress in characterizing the various forms of tissue response to chronic gastritis in humans, the described transitions can only be inferred from available clinical samples because individual lesions cannot be tracked over time within the same patient. For example, intestinal metaplasia almost always accompanies pyloric metaplasia because pyloric metaplasia and oxyntic atrophy are essentially inextricable: loss of parietal cells correlates with more global histological changes, including SPEM (Goldenring and Nam, 2010). In addition, intestinal metaplasia typically does not appear unless oxyntic atrophy (and pyloric metaplasia) is already present (Tong et al., 2024). However, we do not know whether these lesional states exist in an equilibrium or arise sequentially. It seems likely that pyloric metaplasia and oxyntic atrophy are reversible, as they have been shown to be temporary regenerative lesions in animal models (Goldenring, 2018), and are observed to occur spontaneously in small foci in otherwise normal human stomach (Choi et al., 2014). However, whether intestinal metaplasia reverts to pyloric metaplasia and then recurs remains unclear. Without animal models recapitulating these transitions, such questions are difficult to answer. Although pyloric metaplasia with SPEM can easily be modeled in animals in both acute and chronic conditions, inflammation-associated, spontaneously arising intestinal metaplasia has largely not been described in mice (let alone progression of such lesions to adenocarcinoma) despite efforts to model this process (Petersen et al., 2017). Murine models of chronic atrophic gastritis and pyloric metaplasia have, nevertheless, been essential in expanding our understanding of molecular pathways and cell types involved in gastric metaplasia development. Moreover, they can be used to assess the benefits of early intervention and identify early biomarkers for the prevention of gastric carcinogenesis (Hoft et al., 2025). An overview of pre-neoplastic metaplasia models is depicted in Table 1 and discussed in more detail below.

**Table 1.** Summary of gastric preneoplasia mouse models

Category	Advantages	Limitations	Model	References
Drugs	Rapid induction of metaplasia (days to weeks)	Mechanisms of action not fully understood	DMP-777	Nomura et al., 2005b; Goldenring et al., 2000; Ogawa et al., 2006a
			L635	Goldenring et al., 2000; Nam et al., 2010; Petersen et al., 2014
			Tamoxifen	Huh et al., 2012; Saenz et al., 2016; Keeley et al., 2019; Lee et al., 2017; Burkitt et al., 2017b; Bockerstett et al., 2020
Infections	Recapitulates chronic inflammatory process seen in humans	Slow induction of metaplasia (>6 months)	*Helicobacter pylori* SS1	Lee et al., 1997; Arnold et al., 2011a; Serizawa et al., 2016; Sáenz et al., 2019; Fu et al., 2024b
			*Helicobacter felis*	Lee et al., 1990; Fox et al., 1996; Wang et al., 1998
			*Streptococcus anginosus*	Fu et al., 2024b
Immune	Facilitates focused studies on role of immune cells and immune signaling	Difficulty in tuning immune stimulus	Atp4b-Ifnγ	Syu et al., 2012
			*Hip1r* ^−/−^	Jain et al., 2008; Liu et al., 2012
			TxA23	Nguyen et al., 2013
			Krt19-*Il11*	Buzzelli et al., 2019
			Exogenous IL-11	Howlett et al., 2012
			Exogenous IL-33	Buzzelli et al., 2015
Oxyntic atrophy	Induces loss of parietal cells, which is associated with metaplasia	Unable to study effects of interventions on parietal cells	*Atp4a* ^−/−^	Spicer et al., 2000; Judd et al., 2005
			*Atp4b* ^−/−^	Scarff et al., 1999
			Atp4b-DT	Li et al., 1996
Growth factors	Allows for study of role of different signaling pathways	Difficulty in modeling nonautonomous effects and gradients	MT-Tgfα	Goldenring et al., 1996
			Waved-2+DMP-777	Ogawa et al., 2006b
			*Areg* ^−/−^	Nam et al., 2009
			Atp4b-Nog	Shinohara et al., 2010
			Atp4b-Cre; *Bmpr1a^flox/flox^*	Takabayashi et al., 2023
			*Lgr5*^*CreERT2*^; *Bmpr1a^flox/flox^*	Ye et al., 2018
			Six2-Cre; Pkacα	Puri et al., 2022
E-cadherin	Phenotype consistently involves development of signet ring-like cells	May be limited to modeling early lesions of diffuse gastric cancer specifically	Atp4b-Cre; *Cdh1^flox/flox^*	Mimata et al., 2011
			*Mist1*^*CreERT2*^; *Cdh1^flox/flox^*	Hayakawa et al., 2015
			Tff1-Cre; *Cdh1^flox/flox^*	Kinoshita et al., 2019
Glucocorticoids	Allows study of the roles of adrenal hormones	Requires developing proficiency in a surgical procedure	Adrenalectomy (F)	Busada et al., 2019
			Adrenalectomy+castration (M)	Busada et al., 2019
Kras	Models genetic alterations to a commonly mutated oncogene	May not reflect disease process induced by other genetic mutations	Krt19-KrasV12	Brembeck et al., 2003
			*Mist1^CreERT2^*; *Kras^G12D^*	Hayakawa et al., 2015; Choi et al., 2016
			*Lgr5^CreERT2^*; *Kras^G12D^*	Leushacke et al., 2017
			*Gif^rtTA^*; TetO-Cre; *Kras^G12D^*	Won et al., 2024

Cre, Cre recombinase; DT, diphtheria toxin; F, female; IL, interleukin; M, male; MT, metallothionein; rtTA, reverse tetracycline-controlled transactivator; SS1, Sydney strain 1; TetO, tetracycline operator.

### Drug-induced mouse models of gastric preneoplasia

The most biologically accurate models of gastric atrophy and metaplasia are murine models that develop chronic inflammation, mirroring the chronic inflammation-driven metaplasia observed in humans. In mice, such chronic inflammation can be induced by *Helicobacter* spp. infections (Lee et al., 1997; D'Costa et al., 2018) and by induction of autoimmune gastritis (Wang et al., 2026) (Table 1). However, chronic infection models require mice to be housed for prolonged periods of time (many months) before metaplasia arises (Petersen et al., 2017), and the resulting metaplasia is invariably asynchronous across the stomach (Weis et al., 2013).

In contrast, drugs have the advantage of rapid and more synchronous metaplasia induction within days (Petersen et al., 2017). There are three drugs that have been reported to reliably cause rapid parietal cell loss and pyloric metaplasia: DMP-777, L635 and tamoxifen. DMP-777 was originally developed as a neutrophil elastase inhibitor but also acts as a protonophore in parietal cells, which was supported by the observation that DMP-777-induced oxyntic atrophy is ameliorated by omeprazole (a proton pump inhibitor) treatment (Ogawa et al., 2006a). It induces rapid loss of murine parietal cells within 3 days, followed by pyloric metaplasia within 2 weeks of treatment that is reversible 3 months after withdrawal (Goldenring et al., 2000). Neither dysplasia nor neoplasia has been observed to arise in this model (Nomura et al., 2005b). L635 is structurally related to DMP-777 and also functions as a parietal-cell-specific protonophore but does not inhibit neutrophil elastase (Goldenring et al., 2000). It induces oxyntic atrophy accompanied by a robust inflammatory response (which is absent in DMP-777 treatment) and development of pyloric metaplasia within 3 days of treatment (Goldenring et al., 2000), partly mediated by activity of M2 macrophages (Nam et al., 2010; Petersen et al., 2014). Tamoxifen is a selective estrogen receptor modulator, high doses of which also cause rapid parietal cell loss and pyloric metaplasia in mice within 3 days of treatment with little inflammation (Huh et al., 2012; Saenz et al., 2016; Keeley et al., 2019; Lee et al., 2017; Burkitt et al., 2017b; Bockerstett et al., 2020), and the phenotype is reversed by 3 weeks (Huh et al., 2012). However, the oxyntic atrophy and metaplasia do not appear to be related to tamoxifen's estrogen-related activity, as estrogen antagonists do not affect its oxyntic atrophy induction, and other drugs with similar estrogen-modulating effects do not reproduce this phenotype (Huh et al., 2012). Moreover, it has also been shown to act as a protonophore *in vitro* (Manning et al., 2020).

### Bacterial infection-induced mouse models of gastric preneoplasia

*H. pylori* infection is the most common cause of metaplasia leading to the majority of gastric adenocarcinoma cases (Graham, 2015), and its eradication is associated with reduced incidence of gastric cancer (Lee et al., 2016). Extensive research has been conducted to understand the pathogenesis of *H. pylori* (Calam et al., 1997; Testerman and Morris, 2014; Chmiela and Kupcinskas, 2019) to identify therapeutic targets. Such efforts have been greatly facilitated by murine models of *Helicobacter* infection (Duckworth et al., 2015; D'Costa et al., 2018). For example, early studies in mice demonstrated that *H. pylori* expresses adhesins to recognize sialylated glycans on host gastric epithelial cells (Syder et al., 2003; Oh et al., 2006), and preventing this interaction has been shown to be a potential treatment option (Teng et al., 2022; Sáenz et al., 2019). Moreover, these models recapitulate the pathology seen in humans with *H. pylori* infection (Öztekin et al., 2021), including oxyntic atrophy and metaplasia (Burkitt et al., 2017a).

Most *H. pylori* infection models use C57BL/6 mice infected with *H. pylori* pre-mouse Sydney strain 1 (PMSS1) (Lee et al., 1997). PMSS1 is one of the few cagPAI-positive strains successfully adapted for long-term infection in mice (Arnold et al., 2011a). The cagPAI is the pathogenicity island in the genome of some *H. pylori* strains that encodes the CAG toxin, which has multifaceted oncogenic effects upon its injection into gastric epithelial cells (Tran et al., 2024). PMSS1 infection in C57BL/6 mice causes inflammation, oxyntic atrophy and compensatory epithelial hyperplasia within a month (Arnold et al., 2011a). Within 3 months of infection, mice develop acidic mucus-positive metaplasia (Arnold et al., 2011a) accompanied by increased expression of cytokines and SPEM markers, including SOX9 (Serizawa et al., 2016). The drug-induced metaplasia models discussed above can be used to better investigate the influence of metaplastic changes on *H. pylori* pathogenesis because these drugs are able to induce metaplasia synchronously throughout the stomach compared to the asynchronous metaplasia seen in models using bacterial infection alone (Weis et al., 2013). When acute pyloric metaplasia in mice was induced by high-dose tamoxifen, *H. pylori* PMSS1 showed significantly increased colonization of gastric corpus driven by elevated levels of sialyl-Lewis X on the surface of SPEM cells, which provide binding sites for the bacteria (Sáenz et al., 2019). This suggests that it is possible that *H. pylori* expands its niche from the initial colonization of the stomach antrum into the gastric corpus in some patients, increasing the risk of gastric cancer by driving the described epithelial metaplastic changes (Sáenz et al., 2019).

A related species of *Helicobacter* isolated from cats, *Helicobacter felis* (Lee et al., 1990), is used to study *Helicobacter* infection in mice, also inducing the oxyntic atrophy and SPEM (Fox et al., 1996; Wang et al., 1998; Duckworth et al., 2015) that is seen in humans. Studies in this model showed that the host immune response plays an important role in the pathogenesis, given that T-cell-deficient mice do not develop oxyntic atrophy following *Helicobacter* infection (Roth et al., 1999). Similarly, it has been shown that humans experimentally challenged with *H. pylori* develop a robust T-cell response (Nurgalieva et al., 2005) and that T-helper cells promote gastric mucosal inflammation (Talayev et al., 2024). Importantly, intestinal metaplasia and gastric adenocarcinoma have not been observed in these models for unclear reasons.

In recent years, the contribution of non-*H. pylori* microbes to gastric tumorigenesis has gained more recognition. A recent study found that, compared to mice infected with *H. pylori* alone, mice coinfected with *H. pylori* and *Staphylococcus epidermidis* had less proinflammatory cytokine production, and coinfection with *H. pylori* and *Streptococcus salivarius* resulted in more significant gastric pathology (Shen et al., 2022). Sung and Yu's group demonstrated that *Streptococcus anginosus* infection induced progressive chronic gastritis, severe oxyntic atrophy, mucinous metaplasia and low-grade dysplasia 12 months post-infection in C57BL/6 mice (Fu et al., 2024b). In this study, compared to *S. anginosus* infection, *H. pylori* infection resulted in a lower number of mucinous metaplasia cases with no dysplasia observed at 12 months post-infection (Fu et al., 2024b). These results provide evidence that non-*H. pylori* microbes can cause preneoplasia and gastric cancer development, establishing an important direction for further studies.

### Parietal-cell-based mouse models of gastric preneoplasia

In addition to the approaches described above, there have been several genetic engineering approaches that induce parietal cell loss and oxyntic atrophy in mice. Most of these approaches harness the parietal cell specificity of H^+^/K^+^ ATPase, a multimeric enzyme that includes α and β heterodimeric subunits (Shin et al., 2009). An early study in this field showed that promoter elements from the gene encoding the H^+^/K^+^ ATPase β-subunit (*Atp4b*) can be used to drive transgenic expression of diphtheria toxin specifically in parietal cells, leading to their ablation and disruption of gastric epithelial homeostasis (Li et al., 1996). It is important to note that the promoter elements from the *Atp4b* gene drive expression of genes in both parietal cell progenitors and parietal cells [see discussion and single-cell RNA-sequencing data in Adkins-Threats et al. (2024)]. Additionally, mice null for the α or β subunits of this enzyme [i.e. *Atp4a^−/−^* mice (Spicer et al., 2000) or *Atp4b^−/−^* mice (Scarff et al., 1999)] have been shown to have impaired parietal cell function. *Atp4a^−/−^* mice developed achlorhydria from lack of gastric acid production (Spicer et al., 2000), and hypergastrinemia that is known to result from achlorhydria (Fahrenkrug et al., 1976). Although the *Atp4b^−/−^* mice had similar achlorhydria and hypergastrinemia to the *Atp4a^−/−^* mice, only the former showed gastric mucosal hypertrophy (Scarff et al., 1999). Moreover, *Atp4a^−/−^* mice developed ciliated metaplasia (Spicer et al., 2000), which can be associated with gastric carcinoma development in humans (Rubio et al., 2005). Together, these results confirm that parietal cell function is important for maintenance of gastric epithelial homeostasis and that mouse models of impaired parietal cells can be used to investigate gastric tumorigenesis.

### EGFR-driven mouse models of gastric preneoplasia

The epidermal growth factor receptor (EGFR) and its ligands are important in development and tissue homeostasis (Schneider and Wolf, 2009). Dysregulated EGFR signaling can lead to diseases including those caused by overexpression of the EGFR ligand TGFα (e.g. Ménétrier disease) (Huh et al., 2016). Excessive TGFα in the gastric epithelium can stimulate foveolar cell proliferation and inhibit gastric acid secretion (Dempsey et al., 1992). Moreover, patients with this condition have increased risk of developing gastric cancer, likely related to the associated hyperproliferation (Coffey et al., 2007). This phenotype can be reproduced in mice by inducing transgenic *Tgfa* expression under regulation of metallothionein promoter element (Goldenring et al., 1996), for example, through zinc treatment (Nomura et al., 2005a). More recently, a doxycycline-induced Ménétrier model that allows better control of TGFα expression was developed (Gabriel et al., 2025). This is an improvement on the previous model, which was shown to have a leaky promoter resulting in phenotypes even without zinc treatment (Gabriel et al., 2025). An earlier model, the waved-2 mouse model features a mutant EGFR that mimics mice deficient in TGFα (Fowler et al., 1995). Interestingly, when pyloric metaplasia was induced in the waved-2 mouse via DMP-777 treatment, the development of SPEM occurred much faster than in wild-type mice, suggesting that EGFR signaling is important in this process (Ogawa et al., 2006b).

Another EGFR ligand, amphiregulin, is known to promote the growth of human gastric cancer cell lines (Akagi et al., 1995) and may serve as a gastric cancer biomarker, as high amphiregulin expression in gastric cancer biopsies is correlated with poorer prognosis (Wang et al., 2016). Interestingly, *Areg*-deficient mice exhibited pyloric metaplasia development by 18 months of age (Nam et al., 2009), which was accelerated to 2 weeks as would be expected following DMP-777 treatment (Nam et al., 2007). These results provide additional support of the role of amphiregulin and, more broadly, EGFR signaling in promoting pyloric metaplasia. Recent work has further elucidated the role of EGFR signaling via these two ligands in regulating gastric progenitor cell fate in response to mucosal injury. Amphiregulin promotes isthmic progenitor differentiation towards parietal cell phenotype, whereas TGFα promotes surface cell lineage commitment (Lee et al., 2024). These results suggest that different EGFR ligands have distinct effects in the gastric epithelium and that targeting amphiregulin could be a therapeutic approach to recover parietal cells that are typically lost following gastric injury or infection.

### BMP-driven mouse models of gastric preneoplasia

Bone morphogenetic proteins (BMPs) are growth factors in the TGFβ superfamily and are important in development and homeostasis of numerous tissues, including the stomach (Zhang and Que, 2020). Mice with *Atp4b*-driven expression of the BMP inhibitor noggin exhibit oxyntic atrophy (i.e. loss of parietal cells), SPEM and foveolar hyperplasia (Shinohara et al., 2010). The foveolar hyperplasia (but not the other phenotypic changes) was ameliorated by crossing these mice with gastrin-deficient mice (Todisco et al., 2015). These results show that loss of BMP signaling induces SPEM development independently of gastrin, suggesting that BMP could be potentially targeted to prevent gastric metaplasia. BMP signaling has also been shown to have protective anti-inflammatory effects on mouse gastric mucosa, as evidenced by increased *Helicobacter*-induced inflammation in mice with overexpressed noggin (Takabayashi et al., 2014). Thus, BMP may be an especially important therapeutic target in infection-induced gastric metaplasia.

The G-protein-coupled receptor LGR5 is expressed in the stomach antrum in isthmal stem cells and in deep mucous cells (Barker et al., 2010), as well as in chief cells in the corpus (Leushacke et al., 2017). Given its relative specificity as a marker of a subset of cells in the gastric epithelium, *Lgr5* has been used to generate various mouse models of gastric preneoplasia and cancer. Inhibition of BMP signaling through *Lgr5*-specific deletion of the gene encoding the BMP receptor 1A (*Bmpr1a*) in mice, combined with *Helicobacter* infection, led to pyloric metaplasia and gastric dysplasia, with LGR5-positive cells (likely including chief cells) differentiating into SPEM cells (Ye et al., 2018). This provides evidence that chief cells are a source of SPEM and gastric cancer. Parietal-cell-specific knockout of *Bmpr1a* reproduced the phenotype of the transgenic noggin mice by 6 months of age, with altered parietal cell differentiation and acquisition of metaplastic gene signatures (Takabayashi et al., 2023). These results confirm the importance of BMP signaling in maintenance of parietal cell homeostasis. Nonspecific deletion of *Bmpr1a* in the stomach epithelium using CAGG-CreER™ mice, which are mice that have a tamoxifen-inducible Cre recombinase that can mediate deletion of target genes in all tissues, resulted in antral-pyloric polyp formation due to loss of antral BMP signaling (Huh et al., 2010). Interestingly, there was a lack of phenotype observed in the corpus in this model despite loss of corpus BMP signaling (Huh et al., 2010). These results suggest that there may be differences in stomach antrum and corpus dependence on BMP signaling.

Protein kinase A (PKA) generally inhibits BMP activity (Gupta et al., 1999) and promotes mitogen-activated protein kinase (MAPK)/ERK signaling (Stork and Schmitt, 2002). Constitutively, activation of PKA in mesenchymal cells of the mouse stomach via *Six2-Cre* results in downregulated BMP and upregulated ERK signaling, leading to classic features of gastric preneoplasia including oxyntic atrophy, SPEM and invasive glands (Puri et al., 2022). These results are consistent with the observations in the above models in which BMP signaling is inhibited, providing further support for the importance of this pathway in the stomach.

### *Kras*-driven mouse models of gastric preneoplasia

The Kirsten rat sarcoma virus (*KRAS*) oncogene is upregulated in over 40% of intestinal-type human gastric cancers (The Cancer Genome Atlas Research Network, 2014), which has prompted extensive efforts towards generating mouse models of KRAS-driven gastric cancer (Won and Choi, 2022). The KRAS-driven mouse models that have resulted in the development of gastric cancer and metastasis have typically featured additional dysregulation of various tumor suppressors and will be discussed in subsections of the ‘Genetically engineered models of gastric cancer’ section (‘Stem-cell-driven models’, ‘Chief-cell-driven models’ and Pan-epithelium-driven models’). The Rustgi group published an early report on the role of KRAS in the gastric epithelium using Krt19-KrasV12 transgenic mice (Brembeck et al., 2003), in which regulatory elements of the cytokeratin 19 (Krt19) promoter drive expression of a constitutively active mutant *Kras* gene in epithelial cells. These mice developed hyperplasia of mucous neck cells and loss of parietal cells, which are typical features of gastric preneoplasia. It is important to note that Krt19-driven transgenic expression of lacZ (used as a reporter for Krt19 expression) did not have a well-defined expression pattern and included cells from the surface to the base and within the isthmus progenitor cell zone (Brembeck et al., 2001). Thus, it is difficult to determine the gastric epithelial cell type of origin responsible for the phenotypes seen using this model. Another study demonstrated that Krt19-KrasV12 mice exhibited parietal cell loss, metaplasia, dysplasia and papillary tumors (Okumura et al., 2010). This suggests that mutant *Kras* in the mouse stomach can induce features of gastric tumorigenesis, including preneoplastic lesions seen in humans.

Mouse strains such as *Mist1^CreERT2^* express inducible, cell-lineage-specific Cre recombinase, which enables mutant *Kras* induction in a temporal and cell-specific manner (Habbe et al., 2008). The transcription factor MIST1 (also known as BHLHA15) is a terminal differentiation transcription factor that scales up secretory architecture in exocrine secretory cells, including chief cells (Lo et al., 2017), which are a key source of SPEM in humans (Lennerz et al., 2010) and mice (Nam et al., 2010). *Mist1* promoter-specific activation of oncogenic *Kras* in mouse chief cells was shown to result in SPEM with intestinal metaplasia features within 3-4 months of Cre induction, which could be reversed by treating these mice with a Ras signaling inhibitor (Choi et al., 2016). These results suggest that targeting the *Kras* signaling pathway could be beneficial in patients with gastric metaplasia, which has led to recent clinical trials targeting mitogen-activated protein kinase kinases (MAP2Ks; also known as MEKs), a downstream effector of KRAS (Kim et al., 2024).

A conflicting perspective proposes that rare MIST1-expressing isthmus stem cells are the origin of metaplasia in this model (Hayakawa et al., 2015), but 99% of *Mist1^CreERT2^* expression in the stomach is in chief cells with less than 1% in other cell types, including the isthmus stem cells (Sáenz et al., 2022; Willet et al., 2023). Other models of KRAS activation have used markers more specifically expressed in gastric isthmus stem cells and not in the chief cells to distinguish which of the two cell types give rise to the observed phenotypes. These include elements of *Runx1* promoter (eR1) (Matsuo et al., 2017) and LRIG1 (Choi et al., 2019). In both cases, expression of oncogenic *Kras* in gastric isthmus stem cells resulted in foveolar hyperplasia rather than the full pyloric metaplasia phenotype with SPEM cells at the base (Matsuo et al., 2017; Choi et al., 2019). Moreover, a recent mouse model in which oncogenic *Kras* was driven by gastric intrinsic factor, which is specific to chief cells in rodents (Boass and Wilson, 1964; Lorenz and Gordon, 1993), was found to develop SPEM and eventual dysplasia that was dependent on an altered fatty acid metabolism pathway (Won et al., 2024). Together, these results demonstrate that expression of oncogenic *Kras* specifically in chief cells in the mouse stomach can model preneoplastic lesions seen in humans with early-stage gastric cancer and that understanding the mechanisms that drive chief cell differentiation to SPEM will be key to uncovering therapies that prevent development of gastric cancer.

### TP53-driven mouse models of gastric preneoplasia

Although out of scope for this Review, it is worth mentioning that the tumor suppressor TP53 (i.e. P53, encoded by *Trp53* in mouse) is a critical checkpoint regulating the cell program (paligenosis) that converts chief cells into SPEM cells. Therefore, given that SPEM cells have been implicated in gastric intestinal metaplasia and subsequent tumorigenesis (Miao et al., 2020; Brown et al., 2022), TP53 may have an upstream, permissive role in tumorigenesis. Specifically, loss of TP53 increases the risk of uncontrolled, over-proliferative SPEM that may give rise to cell clones susceptible to further mutation through repeated cycles of paligenosis (Jin and Mills, 2019; Cho et al., 2024).

TP53 is mutated in 71% of the gastric cancer subtypes characterized by chromosomal instability, which represents approximately half of the primary gastric adenocarcinomas analyzed by a TCGA project (The Cancer Genome Atlas Research Network, 2014). Similar to *Kras*, *Trp53* inactivation alone in chief cells using either *Mist1^CreERT2^* or *Lgr5^CreERT2^* drivers does not induce gastric preneoplasia, even following 2 years of monitoring (Sethi et al., 2020). A combination of inactivated *Trp53* with environmental exposures in the form of unconjugated bile acid deoxycholate and N-methyl-N-nitrosourea (see Box 2) enabled the development of dysplastic lesions, with rare instances of invasion in the inducible *Mist1^CreERT2^* model but not in the inducible *Lgr5^CreERT2^* model (Sethi et al., 2020). However, it remains unclear whether these results specifically implicate TP53 in the induction of gastric preneoplasia and cancer, as N-methyl-N-nitrosourea itself is a mutagen that induces gastric cancer in wild-type mice (Box 2) (Miao et al., 2021). When coupled with additional genetic alterations (such as *Kras*, *Apc* and *Smad4*), mutant *Trp53* does appear to contribute to the ability of gastric cancer to metastasize in several mouse models that will be discussed in subsections of the ‘Genetically engineered models of gastric cancer’ section (‘Stem-cell-driven models’, ‘Chief-cell-driven models’, ‘Parietal-cell-lineage-driven models’, ‘Pan-epithelium-driven models’ and ‘Other models’).

Box 2. N-methyl-N-nitrosourea-induced models Many genetic models have used N-methyl-N-nitrosourea as a chemical carcinogen to accelerate gastric cancer (Tatematsu et al., 1992; Tatematsu et al., 1993; Yamachika et al., 1998; Yamamoto et al., 2002). This mutagen can be given to mice in drinking water to promote adenocarcinomas in the antrum, along the antrum–corpus border, and on the forestomach–glandular mucosa border (Li et al., 2021). N-methyl-N-nitrosourea is a methylating agent that directly alters nucleotides in DNA and RNA (Swann and Magee, 1968; Golding et al., 1997), producing O6-methylguanines that miscode in replication to cause G-C base pairs to become A-T. It is also capable of making epigenetic modifications on histone proteins (Boffa and Bolognesi, 1985). In these models, it is important to consider differences between the genetic alteration itself and N-methyl-N-nitrosourea in promoting gastric tumorigenesis. N-methyl-N-nitrosourea has variable tumorigenesis induction that can be mouse-strain dependent. For example, BALB/c is the most susceptible of common strains, and C57/B6 is among the least susceptible to adenocarcinoma induction (Yamamoto et al., 2002). Given that the majority of genetic mouse models are generated using the B6 strain, this aspect of N-methyl-N-nitrosourea is important to consider. Nevertheless, N-methyl-N-nitrosourea has enabled better understanding of gastric cancer molecular mechanisms involving TP53 (Yamamoto et al., 2000), NF-κB (Sakamoto et al., 2010), MAPK (Shibata et al., 2008), COX-2 (Leung et al., 2008; Takasu et al., 2008), β-catenin (Takasu et al., 2008), E-cadherin (Humar et al., 2009), RUNX3 (Ito et al., 2011), FBXW7 (Jiang et al., 2017) and DDIT4 (Miao et al., 2021).

### E-cadherin-driven mouse models of gastric preneoplasia

E-cadherin (encoded by *CDH1*) is important in mediating contact between epithelial cells, and loss of its function is often associated with tumor progression (van Roy and Berx, 2008; Mendonsa et al., 2018). The stomach is particularly sensitive to loss of E-cadherin function (Chan, 2006), even though E-cadherin is universally expressed in epithelial cells in every organ. *CDH1* germline polymorphisms can cause a familial gastric cancer syndrome known as hereditary diffuse gastric cancer (Decourtye-Espiard and Guilford, 2023). The penetrance of developing a diffuse, mucin-filled tumor (so-called signet ring carcinoma) is so high in some families that individuals in their 20s already have multiple foci of tumors, requiring prophylactic total gastrectomy (Hansford et al., 2015).

In addition to inherited genetic variants, gastric tumors show acquired *CDH1* mutations that are present within 37% of tumors of the genomically stable gastric cancer subtype (The Cancer Genome Atlas Research Network, 2014). However, these mutations are much less frequent in the other subtypes (The Cancer Genome Atlas Research Network, 2014; Cristescu et al., 2015). Using Atp4b-Cre; *Cdh1^flox/flox^* mice, which allow for deletion of *Cdh1* specifically in parietal cells and their progenitors, the Yuasa group demonstrated that loss of E-cadherin in ATP4B-expressing cells resulted in altered gastric epithelial cell polarity and differentiation with the appearance of signet ring-like cells (Mimata et al., 2011). In this same mouse model, foveolar hyperplasia, pyloric metaplasia (starting at 3 months of age) and invasive-appearing gland morphology (from 24 months) were also observed (Mimata et al., 2011). These changes, however, occurred in cells with intact E-cadherin expression, consistent with the fact that E-cadherin is needed to form glandular structures (Mimata et al., 2011). Although the ATPase β-subunit-expressing cells were shown to be potential precursors to diffuse gastric cancer (Mimata et al., 2011), expression of the *Atp4b* transgene does not prove that parietal cells are a source of gastric cancer because this marker is expressed in parietal cell progenitors that can reroute to other phenotypes if differentiation is blocked (Syder et al., 2004; Adkins-Threats et al., 2024). For example, when Notch signaling was constitutively activated in parietal cells via transgenic *Atp4b*-driven overexpression of Notch1 intracellular domain (NICD1), parietal cell differentiation was blocked, resulting in the parietal cell progenitor cells going on to produce dysplastic adenomas (Kim and Shivdasani, 2011). Interestingly, *Lgr5*-driven overexpression of NICD1 did not result in similar observations, suggesting the importance of the identity and location of stem cells in promoting gastric tumorigenesis (Kim and Shivdasani, 2011).

Other groups used different Cre drivers to delete *Cdh1*, including Tff1-Cre; *Cdh1^flox/flox^* (Kinoshita et al., 2019) and inducible *Mist1^CreERT2^*; *Cdh1^flox/flox^* (Hayakawa et al., 2015), and observed a similar phenotype with signet ring-like cells, which resemble the morphology of signet ring carcinoma cells seen in humans. Importantly, E-cadherin loss alone was not sufficient to induce gastric cancer in mice and required concurrent loss of *Trp53*. When *Cdh1* deletion was combined with deletion of *Trp53* via *Atp4b-Cre*, these double-mutant mice developed metastatic diffuse-type gastric adenocarcinomas from the rerouted parietal cell progenitors, with lesions largely recapitulating the disease process of human diffuse gastric cancer (Shimada et al., 2012). Interestingly, the additional introduction of oncogenic *Kras* in these mice led to histologically mixed-type, invasive gastric tumors with evidence of both intestinal and diffuse gastric adenocarcinomas (Till et al., 2017).

### Inflammation-based mouse models of gastric preneoplasia

As already highlighted in some of the previously discussed preneoplasia models, inflammation and immune cells play important roles in the development and progression of gastric metaplasia. A classic inflammation-based gastric condition in humans is autoimmune gastritis (Feng et al., 2026), in which autoreactive T cells recognize and target one's own parietal cells. Mouse models have also identified several cytokines to be important in contributing to gastric inflammation, including IFNγ and the interleukin (IL)-1 and IL-6 families, of which several activate the GP130/JAK/STAT pathway (Fig. 2).

**Fig. 2.**
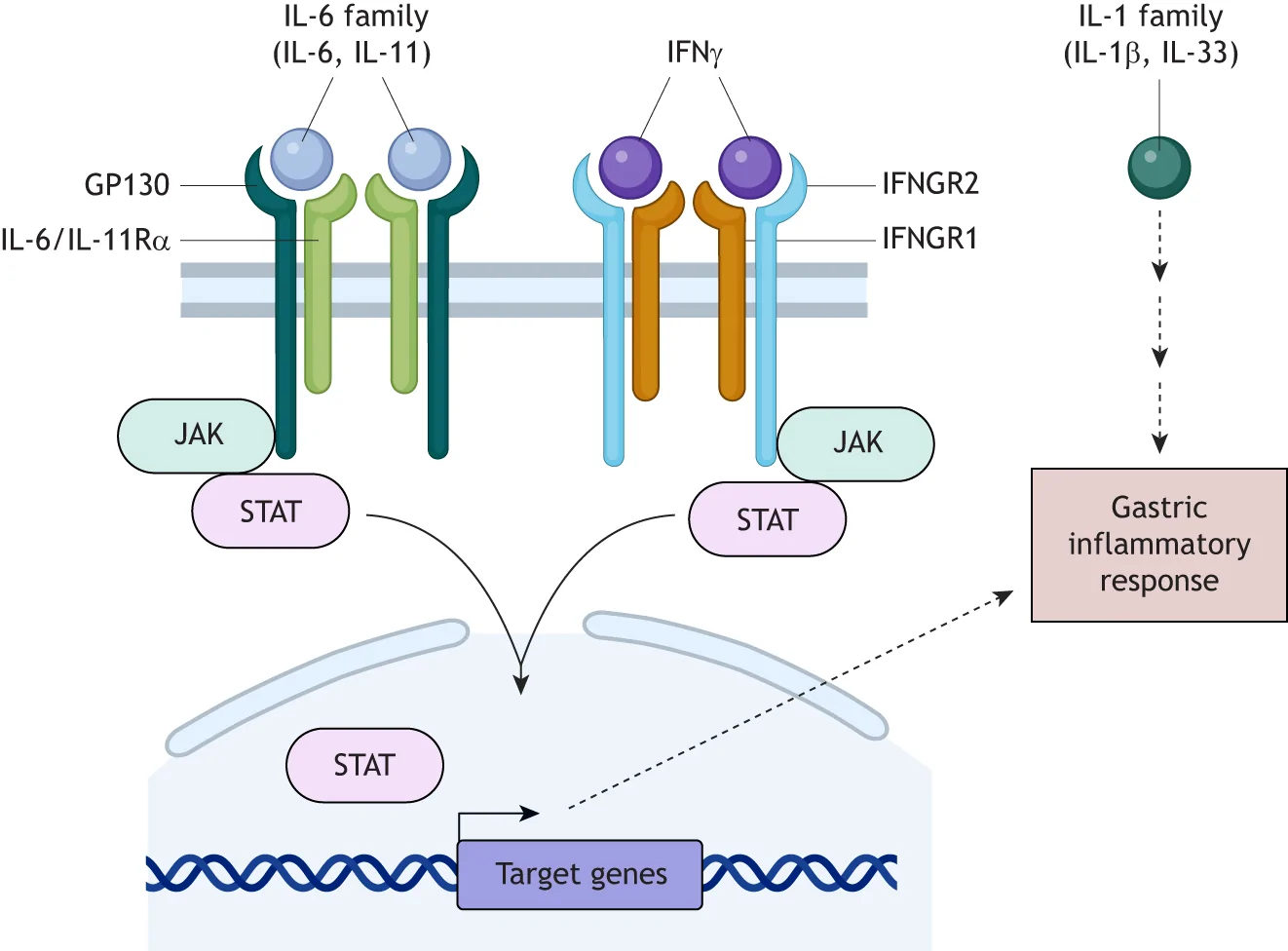
**The GP130/JAK/STAT pathway and cytokines involved in gastric inflammation in mouse models of gastric preneoplasia and cancer.** The IL-6 family of cytokines binds a receptor including the GP130 subunit to initiate JAK/STAT signaling. IFNγ also activates JAK/STAT signaling via its own receptor, independently of GP130. The IL-1 family of cytokines (including IL-33) carries out signaling primarily through NF-κB. All of these cytokines have been shown to play important roles in inducing gastric inflammation. GP130, glycoprotein 130; IFN, interferon; IL, interleukin; JAK, janus kinase; STAT, signal transducer and activator of transcription. Created in BioRender by Zeng, Y. (2026). https://BioRender.com/j3m1mxh. This figure was sublicensed under CC-BY 4.0 terms.

The proinflammatory cytokine interferon IFNγ activates JAK/STAT signaling through binding to its receptor (Ivashkiv, 2018). In mice that overexpress IFNγ in parietal cells, the resulting oxyntic atrophy and pyloric metaplasia are unaffected when the mice are crossed onto a *Rag1* null background (Syu et al., 2012). Mice lacking RAG1 do not have mature T- and B-cell function, so the results indicate that oxyntic atrophy/metaplasia do not require B and T cells. These results suggest that IFNγ is able to induce gastric preneoplasia, with contributions from immune cell types other than lymphocytes, including myeloid cells (Syu et al., 2012), but IFNγ alone is insufficient to cause gastric cancer. TxA23 mice, which model autoimmune gastritis through engineered CD4-positive T-cell recognition of parietal cells, develop oxyntic atrophy, SPEM and extensive IFNγ-dependent inflammation (Nguyen et al., 2013), as demonstrated by rescue of the phenotype upon crossing with IFNγ-deficient mice (Osaki et al., 2019). These results confirm the role of proinflammatory cytokines in promoting gastric preneoplasia development and provide support in the potential efficacy of targeting proinflammatory cytokines in therapeutic approaches.

Other cytokines important in gastric metaplasia development include the ILs (Akdis et al., 2016). In particular, the IL-1 and IL-6 families of cytokines have been highlighted in several mouse models of gastric preneoplasia and cancer. IL-1β transgenic mice develop spontaneous gastric inflammation, with elevated *Il6* and *Tnf* mRNA levels detected at 4 months of age and gastric hyperplasia observed by at least 1 year of age (Tu et al., 2008). IL-11, a member of the IL-6 family of cytokines, is normally produced by parietal cells and induces oxyntic atrophy and SPEM when given exogenously (Howlett et al., 2009, 2012). GP130 (also known as IL6ST) is a receptor subunit that pairs with IL-6/IL-11 receptor subunit α to activate the JAK/STAT pathway after binding interleukins, chiefly IL-6 and IL-11 (Taga and Kishimoto, 1997; Heinrich et al., 1998). Dysregulated JAK/STAT signaling has been shown to contribute to gastric tumorigenesis (Ni et al., 2022), and signal transducer and activator of transcription 3 (STAT3) mediates the early proliferative response in pyloric metaplasia (Khurana et al., 2013). Also, STAT3 deletion in the gastric epithelium suppresses *Helicobacter*-induced inflammation and pyloric metaplasia (Ishii et al., 2018). Interestingly, overexpression of IL-11 through a Krt19 promoter produced similar metaplastic lesions to exogenous IL-11 that were independent of mutations in the GP130/JAK/STAT pathway (Buzzelli et al., 2019). This suggests that IL-11 alone can induce gastric preneoplasia and could be a potential target of therapies using monoclonal antibodies.

IL-33, a member of the IL-1 family of cytokines, is expressed constitutively in surface foveolar cells and can also induce SPEM and inflammation when given exogenously (Buzzelli et al., 2015). These changes are T- or B-cell dependent, as the phenotype can be rescued by crossing with RAG1-deficient mice (Buzzelli et al., 2015). Other mechanistic studies using various murine models identified mast cells (Eissmann et al., 2019), macrophages (de Salvo et al., 2021; Tran et al., 2022), eosinophils (de Salvo et al., 2021) and WAP four-disulfide core domain protein 2 (WFDC2; also known as HE4) (Jeong et al., 2021) as being involved in mediating IL-33-induced metaplasia. IL-33 was also found to upregulate IL-13 signaling in the gastric epithelium, which contributed to pyloric metaplasia development in both murine (Petersen et al., 2018) and metaplastic gastroid (Contreras-Panta et al., 2024) models. The dependence of pyloric metaplasia (with SPEM) on IL-33/IL-13 signaling can be further proved by blocking this pathway in TxA23 mice, which largely prevents metaplasia, even in the presence of chronic inflammation (Noto et al., 2022).

### Glucocorticoid-based mouse models of gastric preneoplasia

The importance of inflammation in promoting gastric metaplasia suggests that a natural defense mechanism against this process likely involves glucocorticoids, which are produced by the adrenal glands and known to have potent anti-inflammatory effects (Coutinho and Chapman, 2011). Recent investigations in the stomach have shown the importance of adrenal hormones using an adrenalectomy mouse model, in which endogenous glucocorticoid levels are depleted (Busada et al., 2019). Such intervention resulted in spontaneous atrophic gastritis and pyloric metaplasia by 2 months of age, mediated by monocytes and macrophages but not lymphocytes (Busada et al., 2019). This phenotype was rescued by administration of the endogenous mouse glucocorticoid corticosterone (Busada et al., 2019). Interestingly, adrenalectomy alone led to the above observations only in female mice, whereas male mice required both adrenalectomy and castration to achieve similar metaplastic lesions, suggesting that androgens have a protective role in the development of gastric preneoplasia (Busada et al., 2021b). Specifically, it was found that the androgen dihydrotestosterone (along with glucocorticoids) is able to both prevent and rescue the gastric metaplasia seen in adrenalectomized female mice by suppressing expression of proinflammatory cytokines by type 2 innate lymphoid cells (ILC2s) (Busada et al., 2021b).

The gastrointestinal tract is protected by a number of anti-inflammatory proteins that can work in conjunction with glucocorticoids (Lima et al., 2022). One such protein, tristetraprolin, is an RNA-binding protein that can be induced by glucocorticoids (Smoak and Cidlowski, 2006) and functions to help degrade proinflammatory mRNAs (Tiedje et al., 2016). In the stomach, overexpression of tristetraprolin prevents the atrophic gastritis and pyloric metaplasia seen in adrenalectomized mice (Busada et al., 2021a). Moreover, inflammation-independent pyloric metaplasia induced by high doses of tamoxifen was unaffected by tristetraprolin overexpression. This result confirms that tristetraprolin's protective effects occur through suppressing inflammation (Busada et al., 2021a). Given that chronic stress accelerates the development of gastric precancerous lesions in mice (Yisireyili et al., 2020) and rats (Zheng et al., 2021), and that prolonged stress in humans leads to glucocorticoid receptor resistance (Cohen et al., 2012), it is likely that this acceleration is mediated by glucocorticoid receptor resistance. This resistance may impair the anti-inflammatory effects of glucocorticoids, exacerbating inflammation and epithelial damage, thereby promoting the progression of preneoplastic gastric lesions.

## Genetically engineered models of gastric cancer

Although murine models of gastric preneoplasia provide important insight into the feasibility of early interventions, given that a majority of patients with gastric cancer present with late-stage disease (Smyth et al., 2020), models of spontaneously arising gastric adenocarcinoma that progress to metastasis are essential for uncovering potential vulnerabilities for therapeutic interventions. The efforts to generate these models build upon the discussed models of preneoplasia using mainly genetic (Table 1), but also some non-genetic engineering strategies. In this section, we focus on genetically engineered murine models of gastric cancer.

### Viral models

Some of the earliest mouse models of gastric cancer leveraged the oncogenic potential of viral proteins. For example, carcinoembryonic antigen-driven expression of DNA oncovirus SV40 (Vilchez and Butel, 2004) resulted in invasive antral tumors from oncogenic SV40 large tumor antigen expression in gastric tissue (Thompson et al., 2000). Mouse mammary tumor virus-driven expression of adenovirus type 12 led to tumor development in the squamocolumnar junction (Koike et al., 1989). Mice with keratin 6-driven expression of human papillomavirus (HPV)-16 early region developed invasive and metastatic tumors from the gastric glandular mucosa (Searle et al., 1994). Expression of SV40 large tumor antigen under control of the *Atp4b* promoter blocked the differentiation of parietal cell progenitors. These cells accumulated with age and, in some mice, transdifferentiated into neuroendocrine cells that formed invasive and widely metastatic ‘small blue cell’ tumors (Syder et al., 2004). These results suggest that different cell types harboring the same oncogene can cause different phenotypes, highlighting the importance of identifying the initial cell type that gave rise to a given gastric cancer. However, these viral and viral gene models do not recapitulate Correa's cascade of progression through oxyntic atrophy and metaplasia. Moreover, the only human gastric cancer associated with viruses is of the EBV etiology, one of the molecular subtypes identified by TCGA (The Cancer Genome Atlas Research Network, 2014). EBV-associated gastric carcinoma (EBVaGC) is a distinct subtype of gastric cancer that originates from a single or a few EBV-infected epithelial cells (Fukayama et al., 1998). The Fukayama group successfully developed a transplantable EBVaGC model, in which a human sample of EBVaGC was inoculated into the backs of severe combined immunodeficiency mice (Iwasaki et al., 1998).

### Gastrin models

Gastrin is normally produced by G cells in the stomach antrum in response to food ingestion and to the state of acid secretion into the stomach lumen. It primarily stimulates gastric acid secretion from parietal cells (Duan et al., 2022). The Ins-gastrin mouse model overexpresses human gastrin via a transgene driven by elements of the insulin gene promoter (Wang et al., 2000). These mice develop oxyntic atrophy (presumably from chronic acid hypersecretion), pyloric metaplasia and dysplastic lesions that resemble carcinoma *in situ* when combined with *Helicobacter* infection (Wang et al., 2000). These results are in line with human studies showing that hypergastrinemia increases the risk of developing gastric cancer (Smith et al., 2017). Mice with β-actin-driven gastrin overexpression developed similar cancer *in situ* in their stomachs, mediated in part by cyclooxygenase-2 (COX-2; also known as PTGS2) (Kanda et al., 2006). COX-2 has been shown to be upregulated in mice infected with *H. pylori* (Xiao et al., 2001), and its increased expression in the stomach is correlated with gastric hypertrophy and metaplasia (Oshima et al., 2004). COX-2 is also highly expressed in human gastric adenocarcinoma (Ristimäki et al., 1997).

Gastrin-deficient mice also show propensity towards metaplasia, polyps and carcinoma *in situ*. One group observed extensive pyloric metaplasia showing focal cells positive for the intestinal marker CDX2 (Saad et al., 2011), indicating onset of intestinal metaplasia as mice aged (Friis-Hansen et al., 2006), which results from the lack of gastric acid (gastrin-deficient mice are achlorhydric) needed to maintain gastric epithelial homeostasis (Dimmler et al., 2003). The achlorhydria also allows for overgrowth of bacteria, which produce carcinogens that contribute to the tumorigenesis seen (Friis-Hansen et al., 2006). Another group observed polyps, extensive pyloric metaplasia and high-grade dysplasia/carcinoma *in situ*, attributed to IFNγ and STAT3 in the epithelial cells (Zavros et al., 2005). However, the results should be interpreted carefully, as mouse stomach anatomy and the ambiguity of Alcian Blue staining can lead to the erroneous identification of invasive cancer and intestinal metaplasia (for more details, see Box 1). To our knowledge, only one paper (Tomita et al., 2011) shows the development of true invasive tumors in gastrin-deficient mice, and this required additional exposure of these mice to the mutagen N-methyl-N-nitrosourea (Box 2). In this case, N-methyl-N-nitrosourea suppressed *Tff1* expression to promote the invasive tumors (Tomita et al., 2011). Overall, gastrin plays an important role in the development of gastric metaplasia and its progression to cancer, resulting in pathology both when present in excess and in its absence.

### Inflammation-based models

As in preneoplasia, inflammation is critical in the development of gastric cancer. For example, polymorphisms of the inflammatory cytokine IL-1β have been associated with increased gastric cancer risk (El-Omar et al., 2001). In a mouse model of *Atp4b* transgene-driven expression of human IL-1β, mice developed atrophic gastritis, SPEM and adenocarcinomas that were accelerated by *Helicobacter* infection and rescued with IL-1 receptor antagonism (Tu et al., 2008). The underlying mechanism is thought to include IL-1β-associated suppression of sonic hedgehog (SHH) signaling in parietal cells (Waghray et al., 2010) and activation of nuclear factor-kappa B (NF-κB) signaling (Tu et al., 2008). Moreover, inhibition of NF-κB, a transcription factor that mediates inflammatory responses (Liu et al., 2017), in mice with IL-1β-induced gastric preneoplasia resulted in improved pathological scores (Tu et al., 2008), suggesting that NF-κB could be a potential therapeutic target. Interestingly, mice deficient in NF-κB1 developed spontaneous exuberant, non-invasive carcinoma-like lesions through upregulated STAT1 and STAT3 activity that was prevented by crossing to STAT1-deficient mice (O'Reilly et al., 2018). Although these results indicate that targeting NF-κB in settings of normal IL-1β signaling may actually promote gastric cancer development, they also confirm that inflammation is important in this process.

As mentioned above, the GP130/JAK/STAT pathway has been shown to play an important role in gastric inflammation (see Fig. 2). A homozygous mouse model in which GP130 is constitutively active via a Y757F knock-in mutation has been shown to develop gastric adenomas by 3 months of age with hyperactivated STAT1/3 signaling (Tebbutt et al., 2002; Jenkins et al., 2005). Further studies identified that dysregulated STAT signaling in these mice causes gastric cancer partly because of upregulated expression of IL-11 (Ernst et al., 2008) and activation of mammalian target of rapamycin complex 1 (mTORC1) (Thiem et al., 2013). Although these studies suggest that targeting STAT is useful in combatting gastric cancer progression, STAT proteins are transcription factors, which are usually hard to target therapeutically (Chen et al., 2026). Thus, identifying other targets involved in gastric inflammation is important for novel therapeutic approaches. For example, mTORC1 was found to be coactivated with STAT3 in both human and mouse gastric tumors (Thiem et al., 2013). Inhibition of mTORC1 in homozygous GP130 mutant mice resulted in suppressed gastric tumor growth (Thiem et al., 2013), suggesting that mTORC1 could be a target for human gastric cancers as well. Heterozygous GP130 mutant mice failed to develop tumors owing to insufficient STAT3 hyperactivation (Jenkins et al., 2005). However, when crossed with transgenic mice with constitutively active hematopoietic cell kinase (Ernst et al., 2002), which is upstream of the GP130/JAK/STAT pathway, heterozygous GP130 mutant mice developed larger gastric adenomas than did homozygous GP130 mutant mice, with foci of invasion-like invagination into submucosa (see Box 1) (Poh et al., 2020). Inhibition of hematopoietic cell kinase in homozygous GP130 mutant mice resulted in reduced tumor size and concomitant reduction in phosphorylated STAT3 compared to that in homozygous GP130 mutant mice with functional hematopoietic cell kinase (Poh et al., 2020). Moreover, increased hematopoietic cell kinase expression was found to be associated with poorer overall survival in patients with gastric cancer (Poh et al., 2020). These findings suggest that hematopoietic cell kinase could be another potential therapeutic target in gastric cancer.

### SMAD models

The SMAD proteins are important mediators of TGFβ signaling (Massagué and Wotton, 2000), with the tumor suppressor SMAD4 acting as a downstream effector (Lagna et al., 1996; Yang et al., 1998). SMAD4 forms stable complexes with SMAD1, SMAD2, SMAD3 and SMAD5, serving as an essential mediator of TGFβ pathway activation (Lagna et al., 1996; Yang et al., 1998). While SMAD4-null mice are embryonically lethal, heterozygous SMAD4 knockout mice develop gastric and duodenal polyps after 1 year of age that resemble human juvenile polyps, characterized by abundant stroma and eosinophilic infiltration, as well as occasional foci of invasive gastric adenocarcinomas (Takaku et al., 1999; Taketo and Takaku, 2000). T-cell-specific SMAD4 deletion has also been shown to be sufficient to induce gastric cancer mediated by abundant proinflammatory cells and cytokines (Hahn et al., 2011; Kim et al., 2006). These mouse models confirm the importance of SMAD4 as a tumor suppressor in the stomach and will serve to help identify novel therapeutic targets. Many patients with gastric cancer have tumors with *SMAD4* deletion mutations (The Cancer Genome Atlas Research Network, 2014), which is associated with poorer survival and reduced responsiveness to therapies (Sun et al., 2025). However, like STAT proteins, SMAD proteins are transcription factors, which makes them difficult to target. A Sleeping Beauty transposon mutagenesis screen, in which transposons are used to unbiasedly activate oncogenes and inactivate tumor suppressor genes (Dupuy et al., 2009), performed in heterozygous *Smad4* mutant mice confirmed that known drivers of human gastric cancer, including *PTEN*, *SMAD4*, *RNF43* and *NF1*, can also induce tumorigenesis in mice. The screen also identified a novel gastric cancer tumor suppressor, LDL receptor-related protein 1B (LRP1B) (Takeda et al., 2016). Thus, such transposon models can help uncover novel targets for gastric cancers resulting from mutations in SMAD4, and the same screening methodology could be applied to other genes.

Mice deficient in SMAD3 developed metastatic colon cancer and proximal gastric lesions with severe infolding and buckling of glands into the submucosa (see Box 1) and expansion of DCLK1-expressing chemosensory tuft cells in those lesions (Zhu et al., 1998; Nam et al., 2012). Recent research has further elucidated the role of these tuft cells in gastric tumorigenesis. In mice, tufts cells were found to promote gastric metaplasia and cancer through releasing IL-25, which activates ILC2s, and targeting of either tuft cells or ILC2s reduced gastric tumor growth (O'Keefe et al., 2023). In patients with gastric cancer, high activity of tuft cells or ILC2s was associated with poorer survival (O'Keefe et al., 2023), suggesting that these therapeutic approaches work in humans as well.

### Stem-cell-driven models

Because cancer is defined as a disease of abnormal proliferation, stem and progenitor cells are seen as a potential origin of cancer owing to their high proliferation ability (Shackleton et al., 2009; Sell, 2010; Chu et al., 2024). To test this hypothesis, significant efforts have been made to develop mouse models of gastric cancer, in which genetic mutations are introduced specifically into gastric stem cells. To date, there are few – if any – markers truly specific to gastric epithelial stem cells (Fig. 3), and none of those are expressed only in the stomach. LGR5 is a marker of crypt-base intestinal cells with stem cell properties (Barker et al., 2007). In stomach, this marker is expressed in several cell types that can exhibit stem cell properties, including a subset of mature chief cells, antral isthmal cells and deep antral mucous cells (Barker et al., 2010). These LGR5-positive cells are thought to act as stem cells for gastric tumors, and many gastric cancer mouse models have used this marker to target genetic drivers. Mice with LGR5-driven deletion of the tumor suppressor adenomatous polyposis coli (*Apc*) develop antral polyps (Barker et al., 2010). LGR5-driven overexpression of GLI2A, a component of the SHH signaling pathway (Sasaki et al., 1999), causes polypoid hyperplastic lesions with submucosal invagination (see Box 1) involving mTORC1 signaling (Syu et al., 2016). Further, LGR5-driven deletion of *Smad4* and *Pten* causes gastric antral tumors in mice that progress to invasive intestinal-type gastric cancer (Li et al., 2016). These results confirm that genes that are known gastric tumor suppressors in humans are sufficient to work as tumor suppressors in mice, even without the other mutations and genome alterations that characterize tumors in humans (The Cancer Genome Atlas Research Network, 2014). Moreover, these findings show that mutations in gastric LGR5-positive cells can cause gastric cancer and that targeting these cells could be a therapeutic approach.

**Fig. 3.**
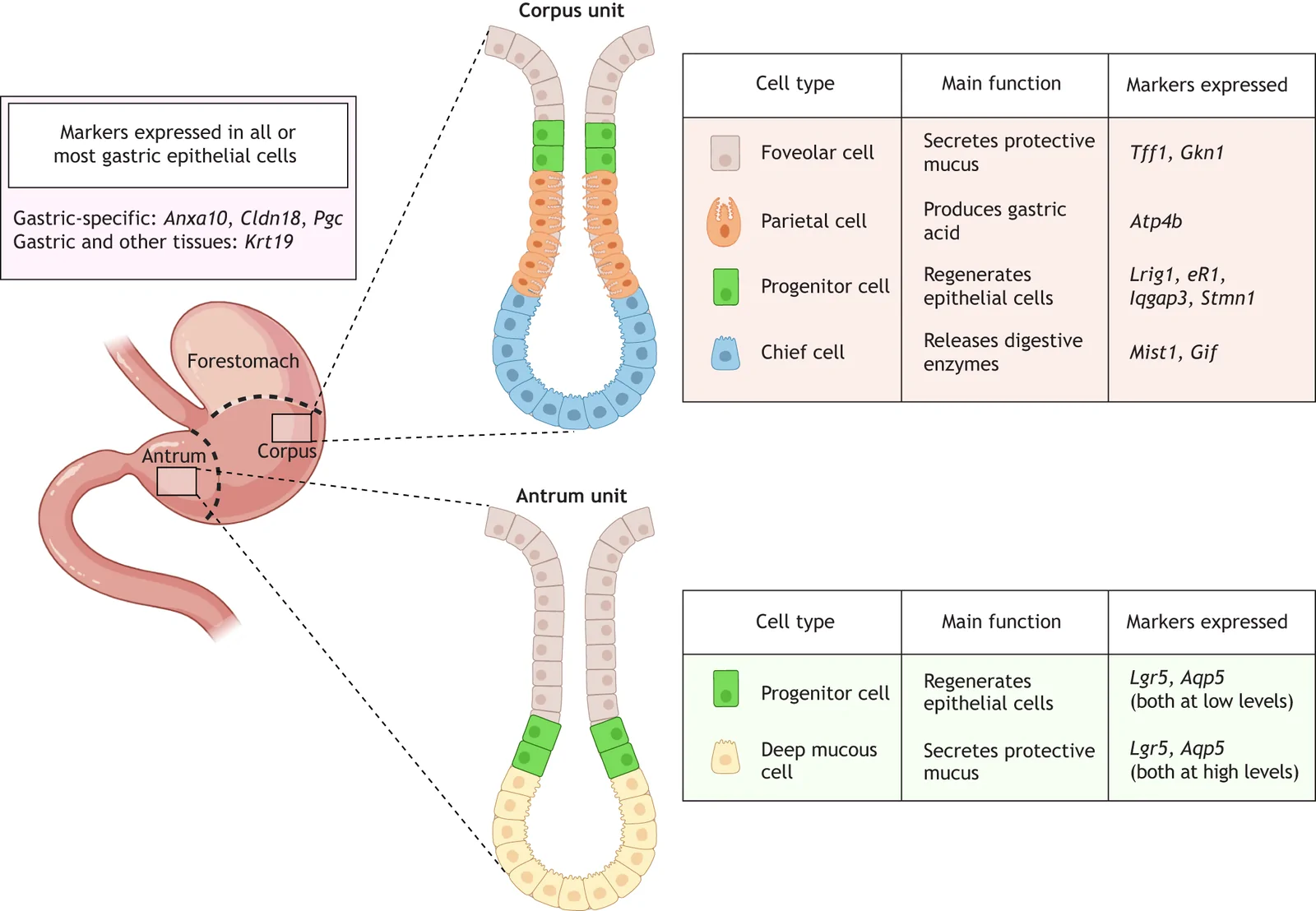
**Commonly used markers in mouse models of gastric preneoplasia and cancer.** The glandular portion of the mouse stomach is divided into the corpus and antrum. The corpus units are largely made up of foveolar cells at the surface, stem cells at the isthmus, parietal cells in the neck and chief cells at the base. In contrast, the antral units are composed of foveolar cells, which make up the majority of each unit, stem cells and deep mucous cells. Note that other rarer cell types, such as endocrine and tuft cells, are present in both the corpus and antrum but are not shown. The key describes the main functions of each depicted gastric cell type, as well as cell-type-specific and nonspecific markers used to generate mouse models of gastric preneoplasia and cancer. Note that mice have a prominent forestomach whereas humans do not. In addition, human antral units can contain some parietal cells and scattered chief cells. *Anxa10*, annexin A10; *Aqp5*, aquaporin 5; *Atp4b*, ATPase, H^+^/K^+^ exchanging, beta polypeptide; *Cldn18*, claudin 18; *eR1*, elements of *Runx1* promoter; *Gif*, gastric intrinsic factor; *Gkn1*, gastrokine 1; *Iqgap3*, IQ motif containing GTPase activating protein 3; *Krt19*, cytokeratin 19; *Lgr5*, leucine rich repeat containing G protein coupled receptor 5; *Lrig1*, leucine-rich repeats and immunoglobulin-like domains 1; *Mist1*, muscle, intestine and stomach expression 1; *Pgc*, pepsinogen C; *Stmn1*, stathmin 1; *Tff1*, trefoil factor 1. Created in BioRender by Zeng, Y. (2026). https://BioRender.com/97if50c. This figure was sublicensed under CC-BY 4.0 terms.

The SOX family of transcription factors plays important roles in development and regulation of stem cell fate (Sarkar and Hochedlinger, 2013). SOX2 is expressed in the adult stem cells of many tissues including in gastric stem cells of the antrum (Arnold et al., 2011b). *Apc* deletion via inducible *Sox2^CreERT2^* led to the development of adenomas that were increased in quantity upon crossing with such SOX2-deficient mice (Sarkar et al., 2016). These results suggest a protective role of SOX2 against gastric cancer progression in the context of dysregulated WNT signaling secondary to *Apc* loss (Sarkar et al., 2016). However, this protective effect was not confirmed in humans, as human adenocarcinomas with gastric differentiation are SOX2 positive (Tsukamoto et al., 2005), particularly in CDX2-induced gastric tumors, which are restricted to the chromosomal instability (CIN) gastric cancer subtype (Voutsadakis, 2025). In addition, SOX2 did not have prognostic value (Voutsadakis, 2025). Thus, SOX2 may play a protective role in certain subtypes of gastric cancer; however, it is not a global tumor suppressor [see discussion in Jin et al. (2025)]. Another member of this family that was found to be expressed in gastric stem and progenitor cells is SOX9 (Chen et al., 2023; Willet et al., 2023). A mouse pedigree featuring *Kras* and *Trp53* mutations introduced into gastric stem and progenitor cells positive for both SOX2 and SOX9 resulted in invasive gastric adenocarcinoma, with lymph and liver metastases, which were prevented by deletion of SOX9 (Chen et al., 2023). These metastases are similar to those seen in patients with advanced gastric cancer (Lauren, 1965). Moreover, patients with high SOX9 expression have poorer prognosis (Chen et al., 2023), suggesting that SOX9 may be worth targeting therapeutically.

AQP5 was recently identified as a gastric stem cell marker in LGR5-positive pyloric stem cells in both mice and humans (Tan et al., 2020). Mice with AQP5-driven expression of oncogenic *Kras* and deletion of *Apc* and *Pten* developed severe hyperplasia with foci of invasion-like infoldings and carcinoma *in situ* at the pylorus (Tan et al., 2020). These findings show that AQP5-positive cells can give rise to gastric cancer when induced with mutations in common genetic drivers. High AQP5 expression has been found in numerous human cancers, including cancers of the gastrointestinal tract, breast and lung (Direito et al., 2016), suggesting that targeting tumor cells expressing AQP5 could be an effective therapy. Although AQP5 can be expressed in cells of the stomach antrum that acquire stem-like behavior, it is also a marker of SPEM, which can arise from LGR5-positive chief cells (Lee et al., 2022). Thus, it is possible that these gastric AQP5-positive cells that give rise to cancer were originally mature chief cells rather than stem cells.

Overall, it is important to remember that no genes have been identified that are expressed exclusively in isthmal, multipotent, undifferentiated stem cells and that cannot also be induced in other cell types (e.g. chief cells and basal antral mucous cells). Thus, we can argue that cell plasticity within the gastric epithelium, especially in the context of injury or inflammation, makes it challenging to determine whether a tumor cell originates from a stem cell or has been recruited as a stem cell from a differentiated cell. For a more detailed discussion of reasons why differentiated cells – with their longer lifespans, less accurate DNA damage repair mechanisms and capacity for multiple rounds of paligenosis that can store genomic alterations – are more likely tumor originators, see review by Cho et al. (2024).

### Chief-cell-driven models

Another hypothesis, supported by a large amount of evidence, suggests that cancer originates from mature, differentiated cells rather than stem cells. Indeed, as most adult organs lack active stem cells, this would be the possible route for tumors such as pancreatic adenocarcinoma (Cho et al., 2024). In the stomach, mature chief cells have been shown to be a key source of metaplasia in infection-induced (Goldenring and Nam, 2010), injury-induced (Mills and Sansom, 2015; Mills and Goldenring, 2017) and mutation-induced mouse models of preneoplasia (Nozaki et al., 2008; Lennerz et al., 2010; Choi et al., 2016, 2019; Won et al., 2024). However, not many mouse models of gastric cancer with the initiating tumorigenic event originating in chief cells exist, partly due to the necessity for multiple genetic mutations to facilitate the progression of metaplasia into cancer. For example, a mouse model that used the chief-cell marker pepsinogen C (PGC) to drive expression of oncogenic *Kras* and deletion of *Apc* and *Trp53* resulted in the development of invasive and metastatic gastric adenocarcinoma (Douchi et al., 2021). However, PGC is not a chief-cell-specific marker, and *Pgc* mRNA is detectable throughout the gastric unit, so its promoter may drive Cre recombinase in non-chief cells (Douchi et al., 2021). Nevertheless, the results here support the theory that multiple genetic alterations may be needed to induce gastric cancer, given that oncogenic *Kras* alone and oncogenic *Kras* with *Apc* deletion both failed to induce invasive gastric cancer (Douchi et al., 2021). This is consistent with the fact that many subtypes of human gastric cancer are defined by multiple genetic alterations (The Cancer Genome Atlas Research Network, 2014; Cristescu et al., 2015).

### Parietal-cell-lineage-driven models

Some studies suggest that mature parietal cells can be a significant source of gastric cancer, but we do not think the existing data support this hypothesis (see discussion in previous subsections: ‘Parietal-cell-based mouse models of gastric preneoplasia’ and ‘E-cadherin-driven mouse models of gastric preneoplasia’). Many of these studies use the discussed *Atp4b* model (Li et al., 1996) to drive transgene expression. For example, mice with *Atp4b*-driven overexpression of NICD1 and deletion of *Trp53* and *Arid1a*, a component of the SWI/SNF chromatin-remodeling complex (Mittal and Roberts, 2020) and one of the most significantly mutated genes in gastric cancer (The Cancer Genome Atlas Research Network, 2014), developed invasive gastric tumors (Loe et al., 2021). However, because transgenes driven by *Atp4b* promoter elements can be expressed by proliferating parietal cell progenitors, it is likely that mature parietal cells are not the source of the tumors seen in these models. Thus, there is, as of yet, no evidence that mature, post-mitotic parietal cells can give rise to tumors.

### Pan-epithelium-driven models

Genes expressed broadly in the stomach epithelium rather than in specific gastric cell types have also been used to generate mouse models of gastric cancer. The Krt19 promoter, as mentioned above, has been used to drive transgene expression nonspecifically in the gastric epithelium (Brembeck et al., 2001). Krt19-Wnt1/C2mE mice are transgenic mice with Krt19-driven overexpression of *Wnt1,* prostaglandin-endoperoxide synthase 2 (*PTGS2*; also known as COX-2) and prostaglandin E synthase (*PTGES*) (Oshima et al., 2006). These mice developed dysplastic gastric polypoid lesions with invasion-like foci and substantial increases in macrophages by 20 weeks of age, with mucous cell metaplasia noted as early as 5 weeks before tumor development (Oshima et al., 2006; Itadani et al., 2009). When genes encoding COX-2 (PTGS2) and mPGES-1 (officially, PTGES, the enzyme that produces PGE2) were additionally overexpressed in these mice, the developed lesions progressed to a more dysplastic appearance by 20 weeks of age, with mucous cell metaplasia noted as early as 5 weeks before tumor development (Oshima et al., 2006; Itadani et al., 2009). Furthermore, lesion growth was significantly suppressed in Krt19-Wnt1/C2mE mice lacking TNF-α (*Tnf^−/−^*) or TNFR1 (*Tnfrsf1a^−/−^*), indicating that TNF-α signaling via TNFR1 plays an important role in gastric tumor development (Oshima et al., 2014).

The actin-bundling protein Villin (also known as VIL1) has been shown to be expressed in the intestines, colon and, upon inflammation, stomach antrum (Braunstein et al., 2002; Qiao et al., 2007). KLF4 is a transcription factor that mediates proinflammatory responses (Feinberg et al., 2005) and is downregulated in human gastric cancers (Wei et al., 2005). Villin-specific deletion of *Klf4* in mice resulted in the development of antral gastric tumors, the incidence of which increased after additional treatment with N-methyl-N-nitrosourea (Li et al., 2012). These results confirm the protective role of *Klf4* in preventing gastric tumorigenesis and suggest it to be a potential therapeutic target. Mutations in peroxisome proliferator-activated receptor delta (PPARD) have been linked to increased gastric cancer risk in humans (Jeon et al., 2013), and PPARD expression is increased in human and mouse gastric tissue infected with *H. pylori* (Nagy et al., 2011). In mice with overexpression of *Ppard* via *Villin-Cre*, invasive gastric adenocarcinomas were observed in the stomach corpus, facilitated by upregulation of IFNγ signaling (Zuo et al., 2019). These findings uncover the mechanism by which PPARD drives gastric tumorigenesis and again highlights the importance of proinflammatory cytokines in this process.

Annexin A10 (ANXA10) was recently identified as a stomach-epithelium-specific protein, and mice with a tamoxifen-inducible CRE (*Anxa10^CreERT2^*) (Seidlitz et al., 2019) have been used to generate mouse models of human gastric cancer subtypes based on the TCGA classification (The Cancer Genome Atlas Research Network, 2014). Specifically, the chromosomal instability subtype was modeled by *Anxa10*-driven expression of mutant *Kras*, *Trp53* and *Smad4*, with the resulting tumors featuring intestinal cancer morphology (Seidlitz et al., 2019). The genomically stable subtype was modeled by two mouse pedigrees: one with mutations in *Kras*, *Cdh1* and *Smad4* that resembled diffuse-type gastric cancer with classic signet ring cells, the other with mutations in *Kras*, *Cdh1* and *Apc* that showed serrated, adenomatous-like lesions on histology (Seidlitz et al., 2019). The histopathology seen in these mouse models closely resembles those seen in the corresponding human gastric cancer subtypes. Therefore, these models have many potential applications, including stepwise mechanistic studies as each gastric cancer subtype progresses, clinical treatment response monitoring and drug screening for each given subtype.

The tight junction protein claudin 18 (CLDN18) is highly expressed in the stomach epithelium (Niimi et al., 2001) and in the lungs (Zhou et al., 2018). Mice with *Cldn18*-driven expression of mutant *Kras*, *Trp53* and *Apc* developed metastatic gastric adenocarcinomas that harbored LGR5-positive cells (Fatehullah et al., 2021). Conventional chemotherapy (5-fluorouracil), which kills rapidly dividing cells, reduced metastases in half of these mice, whereas those receiving a combination of 5-fluorouracil with genetic ablation of the LGR5-positive cells did not develop any metastases (Fatehullah et al., 2021). This suggests that standard chemotherapy treatment regimens for patients with advanced gastric cancer may see benefit from additional specific targeting of LGR5-positive gastric cells. Interestingly, *Cldn18*-deficient mice show disrupted cellular interactions that result in spontaneous gastric tumors with areas of invagination resembling invasion (Hagen et al., 2018). These results indicate that CLDN18 has a tumor suppressor role in the gastric epithelium of mice. In humans, it has been shown that CLDN18 becomes downregulated as gastric cancer progresses, but its prognostic value is weak (Mathias-Machado et al., 2024). Moreover, some patients with metastatic gastric cancer maintain expression of *CLDN18.2*, a splice variant of *CLDN18*, which has become a promising target in recent clinical trials (Shah et al., 2023). Thus, further studies are needed to better understand the role of CLDN18 in the progression of advanced gastric cancer in humans.

### Other models

Other genetically engineered mouse models of gastric cancer include models that use unique genetic mutations or promoters that do not fall into the previous categories. For example, mice with constitutively active dioxin/aryl hydrocarbon receptor developed spontaneous gastric tumors by 12 months of age (Andersson et al., 2002), and this receptor has been found to be increased in patients with gastric cancer (Perrot-Applanat et al., 2024). Mice deficient in the gene *Mth1* (also known as *Nudt1*), which encodes an enzyme important for reducing oxidative stress-induced DNA damage, developed tumors in multiple organs, including the stomach, by 18 months of age (Tsuzuki et al., 2001). In humans, *MTH1* is highly expressed in gastric cancers, and inhibitors targeting this enzyme demonstrated reduced growth of xenograft tumors (Zhou et al., 2019). TFF1 is expressed in foveolar cells of the stomach (Aihara et al., 2017). TFF1-deficient mice were found to develop dysplasia with focal carcinoma *in situ* by 5 months of age (Lefebvre et al., 1996). Recent mechanistic insights into the functions of TFF1 have uncovered that it suppresses gastric tumorigenesis by activating *Trp53* and downregulating STAT3 signaling (Soutto et al., 2014, 2019). These results are also consistent with N-methyl-N-nitrosourea (Box 2) treatment decreasing TFF1 expression, leading to increased tumorigenesis as described earlier (Tomita et al., 2011). Huber et al. (2024) used a Tff1-CreERT2 mouse to drive either gain-of-function mutant *Trp53* or loss of *Trp53*, along with induction of mutant *Kras* and *Pik3ca*, to induce metastatic gastric adenocarcinomas. The development of metastases depended on switches from the metaplastic and early cancer reliance on IL-11 to IL-6 and STAT3 signaling (Huber et al., 2024). A surprising model of gastric tumorigenesis was developed by using a Cre allele based on a constant region of the immunoglobulin gamma1 gene (*Cγ1*^*Cre*^), which was designed for conditional deletion of B-cell genes (Casola et al., 2006). Unexpectedly, this pedigree exhibited unintended expression in gastric neuroendocrine cells, and expression of *Myc* in these cells led to metastatic gastric neuroendocrine carcinomas (Griger et al., 2023). This model might help uncover novel mechanisms of disease development for patients with rare gastric neuroendocrine cancer, for which there are currently no available targeted therapies (Stelwagen et al., 2021).

## Non-genetically engineered models of gastric cancer

Although significantly fewer in number, non-genetically engineered murine models of gastric cancer have several advantages over genetically engineered models. These include a more rapid time course for gastric tumorigenesis, no need for crossing multiple mouse strains, and, in some cases, reliable and reproducible development of the clinically important manifestation of metastatic disease. In addition to a common N-methyl-N-nitrosourea-induced model (Box 2), here, we briefly review some recently developed models that may offer mechanistic insights not easily obtainable from traditional genetically engineered mouse models of gastric cancer.

### Orthotopic models

Orthotopic mouse models of cancer are alternatives to traditional genetically engineered mouse models for both elucidation of the molecular mechanisms of tumorigenesis and testing the efficacy of novel cancer therapeutics (Day et al., 2015). In these models, organ-specific tumors are implanted into the corresponding organ of a host mouse, allowing incorporation of tumors derived from diverse sources, including mice and human patients. Orthotopic implantation of intact tissue isolated from human gastric cancer into nude mice resulted in the development of metastases in 100% of implanted mice compared to the 6.7% occurrence of metastases when implanting disaggregated cell suspensions of the same tissue (Furukawa et al., 1993). Syngeneic orthotopic transplant models involve the implantation of gastric cancer tissue into immunocompetent mice of the same genetic background, allowing for better investigation of the role of immune cells in tumor progression (Shan et al., 2011). Real-time monitoring of primary tumor growth and metastasis in these models has been facilitated by the use of luciferase-tagged gastric cancer cell lines (Busuttil et al., 2018; Giraud et al., 2019). Another study optimized how to generate organoids from patient samples and use them to screen the efficacy of drugs to guide individualized treatment regimens (Steele et al., 2019). Orthotopic models have revealed that the frequently dysregulated activity of GTPase RhoA promotes carcinogenesis in diffuse gastric cancer tumors via focal adhesion kinase (Zhang et al., 2020), identifying it as a potential therapeutic target. Fatehullah et al. (2021) used orthotopic cancer organoids to reveal the critical role of tumor-resident LGR5-positive cells in driving gastric cancer progression and liver metastases. Moreover, a recently developed orthotopic model featuring the use of nanocarbon particles will better enable investigations into how gastric cancer metastasizes to lymph nodes (Fu et al., 2024a). These nanocarbon particles, which are visible to the naked eye upon gross dissection, are injected into the serosa layer of the mouse stomach, and lymphatic drainage can be tracked by following where these particles flowed. Likewise, orthotopic mouse models of gastric cancer peritoneal metastasis have also been described (Li et al., 2023). Because peritoneal metastases are a frequent occurrence and a leading cause of death for patients at this stage of the disease (Manzanedo et al., 2023), such models may be useful for uncovering treatments for patients with peritoneal spread of tumors.

### Somatic model

There have also been recent efforts to develop somatic mouse models of gastric cancer, which are based on introduction of mutations into somatic cells (versus germline-dependent genetic models discussed above). In one study, investigators triggered genetic modifications in the stomach of adult mice via electroporation (Leibold et al., 2024). This novel somatic model offers numerous advantages over traditional genetic models, including the ability to rapidly generate tumor-bearing mice with specific genetic modifications of interest for preclinical studies (Leibold et al., 2024). For example, to model the microsatellite instability subtype of gastric cancer, the researchers introduced mutations to genes critical for DNA repair, *Trp53* and *Msh2*, which resulted in recapitulation of the corresponding disease process seen in humans, allowing further investigations into therapeutic discoveries (Leibold et al., 2024). Importantly, the model also avoids consequences associated with nonspecific gene expression changes during embryogenesis and gastric metaplasia that can be caused by tamoxifen toxicity in traditional genetic models that require tamoxifen-binding Cre recombinase constructs (Huh et al., 2012). However, this model does have several limitations, including the inability to control which gastric cell types receive the somatic alterations from the electroporation method. Thus, studies seeking to investigate the contribution of specific cell types to gastric tumorigenesis would benefit from the genetically engineered models above.

## Conclusions and future perspectives

Murine models of gastric preneoplasia and cancer have significantly improved our understanding of the mechanisms behind gastric tumorigenesis. Preneoplasia models have shown that induction of gastric metaplasia involves loss of parietal cells and chief cells that fuel SPEM in the setting of oxyntic atrophy and pyloric metaplasia. Such models have been elucidating not just the epithelial-intrinsic changes during carcinogenesis but also the influence of inflammatory signaling and stromal cell types on gastric cancer development and progression. In mice, the challenge has historically been in uncovering key regulators of intestinal metaplasia (which mice seem markedly resistant to) and, most importantly, the transition from metaplastic lesions towards the development of gastric adenocarcinomas and metastases. The high genetic heterogeneity of gastric tumors in humans makes them difficult to treat and challenging to model in mice at every stage of cancer progression.

Gastric tumors originate from the epithelial compartment of the stomach, but the specific cell type of origin has been debated. Mouse models of preneoplasia and cancer have suggested isthmal stem cells of antrum or corpus, parietal cell progenitors and chief cells as potentially being the initiating cell types capable of giving rise to gastric cancer, and development of these models has been facilitated by the identification of cell-type-specific markers (Fig. 3). Much of the controversy and confusion with mouse models stems from use of genetic lineage tracing. For example, investigators drive transgene expression using ∼1 kb of the upstream promoter of *Atp4b* and assume that the resulting tumors are due to parietal cell dedifferentiation. Although it is true that those promoter elements typically drive expression restricted to parietal cell progenitors and mature parietal cells, it is important that the *Atp4b* gene (and its corresponding transgenic version) is also expressed in parietal progenitor cells, which are still capable of dividing. If further differentiation into mature parietal cells is blocked, the parietal progenitor cells can reroute to other lineages (Adkins-Threats et al., 2024). Thus, as one of the earliest studies using this transgene showed (Syder et al., 2004), the presence of tumors driven by this promoter does not mean that human, or even mouse, tumors develop from mature parietal cells, which have never been shown to dedifferentiate or divide *in vivo*.

Another area of confusion is related to the possibility of chief cells being the origin of metaplasia and cancer. The confusion stems from the fact that the chief-cell-based promoters can be expressed, at lower levels, in other cell types. For example, although *Mist1* is considered the most chief-cell-specific gene, it is also expressed in pre-zymogenic cells (Ramsey et al., 2007). In addition, some investigators have reported expression of reporter constructs (i.e. to induce fluorescent labeling) driven by the *Mist1^CreERT2^* in the isthmus and progenitor zones and have interpreted these results as evidence that *Mist1* can mark stem cells. However, endogenous MIST1 protein has never been shown to be expressed outside the chief cell lineage. The administration of tamoxifen to activate the *Mist1^CreERT2^* model further complicates matters. Tamoxifen can induce parietal cell death and chief cell conversion to SPEM, resulting in rapid loss of *Mist1* expression, potentially confounding lineage-tracing studies aimed at tracking the fate of the original *Mist1*-expressing cells. Many of these questions, however, may be purely academic exercises, as there is clear, marked plasticity in the gastric epithelium, and gastric epithelial cells can switch fates after injury to facilitate repair. Thus, even if a tumor is proved to originate from an isthmal stem cell, the stem cell itself could have derived a few days earlier from a chief cell (Cho et al., 2024).

Overall, the mouse models have illustrated and further highlighted how gastric neoplastic morphologies and invasive or metastatic behaviors can arise from both intrinsic factors, such as key gene mutations, and extrinsic factors, including *H. pylori* and EBV infections. Although gastric carcinogenesis has been extensively studied, an underlying aspect of tumorigenesis – the development of pyloric metaplasia/preneoplasia under mildly abnormal conditions such as chronic inflammation – remains largely understudied. Paligenosis mouse models provide valuable tools for investigating the origins of gastric preneoplasia and the factors that determine whether it progresses toward regeneration or tumorigenesis. There is perhaps much to be learned by studying paligenosis, as this process enables mature cells to undergo multiple rounds of cell division and redifferentiation, potentially increasing tumorigenic risk through accumulation of cyclical mutations in long-lived cells (Jin and Mills, 2019; Cho et al., 2024). Furthermore, when cancers arise from faulty paligenosis, it is possible that the mature tumor cells retain the capacity to divide by paligenotic mechanisms, rather than stem-cell-like mechanisms, to fuel tumor growth. Given that paligenosis has so far been modeled only in animals, rather than *in vitro*, mouse models will continue to be useful in developing therapeutics to encourage regenerative and discourage tumorigenic paligenosis.

Future mouse models of gastric carcinogenesis must go beyond intrinsic processes, such as paligenosis, and should also accurately reflect extrinsic factors involved in gastric cancer initiation. Moreover, because metastases are the primary cause of cancer-related deaths, developing reliable metastatic gastric cancer models will be critical for identifying new therapeutic targets. To date, most existing mouse models fail to progress to metastatic disease in a way that allows for detailed mechanistic studies. In fact, a large number of papers interpret polyps and other lesions with exuberant hyperplasia and focal dysplasia or carcinoma *in situ* as invasive cancer (Box 1, Fig. 4). A recently developed somatic model of tumorigenesis has shown promise, as it consistently produces metastases and enables investigations into metastatic organotropism. In sum, the field continues to need additional models and more extensive characterization of existing models to better capture the full progression of gastric cancer, including metastasis. Expanding and enhancing the translatability of these mouse models will be essential for developing effective, long-lasting therapies.

**Fig. 4.**
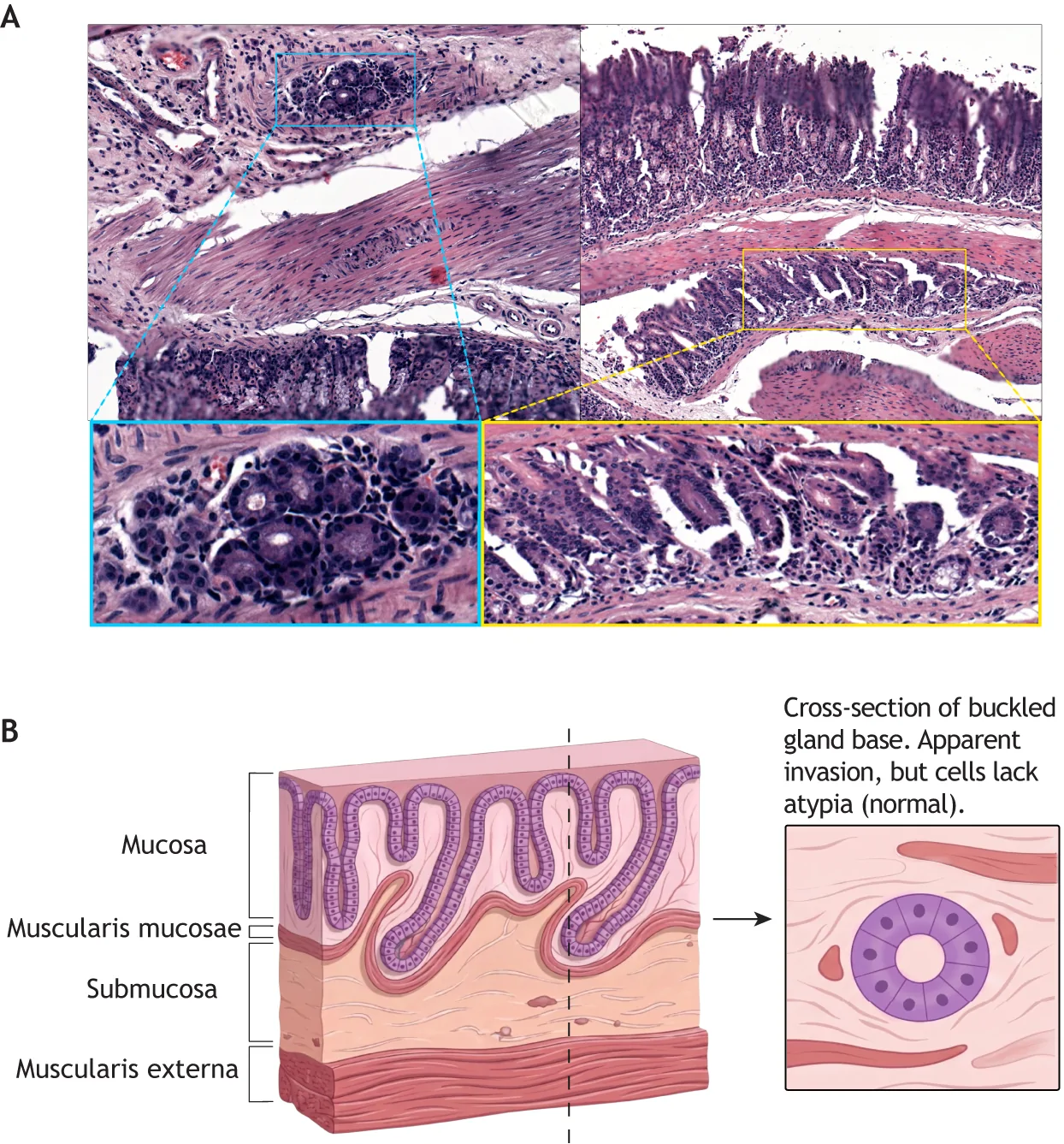
**Examples of pseudo-invasive foci in mouse stomach.** (A) Hematoxylin and Eosin staining of two different sections of mouse gastric tissue. The gastric glands are hyperplastic, and there appear to be foci of invasion (blue and yellow boxes). Magnified views of these foci, however, show that the glands are symmetrical and well ordered, and the cells lack any atypia. These pseudo-invasive foci are the result of either buckling and folding of the mucosa and/or because hyperplastic glands can disrupt the thin murine muscularis mucosa and submucosa. Such foci are frequent in the setting of hyperplastic mucosa and should not be interpreted as invasive cancer. Representative images are derived from source data originally described in Nguyen et al. (2013). (B) Schematic illustration of how pseudo-invasion might manifest in mouse gastric glands. Cells in cross-section (dashed line) of pseudo-invasion regions, which appear to have an invaginated gland base buckling the muscularis mucosa and lamina propria, lack atypia. Created in BioRender by Zeng, Y. (2026). https://BioRender.com/sg283l5. This figure was sublicensed under CC-BY 4.0 terms.
